# A Multiple Antigen Peptide Vaccine Containing CD4^+^ T Cell Epitopes Enhances Humoral Immunity against *Trichinella spiralis* Infection in Mice

**DOI:** 10.1155/2020/2074803

**Published:** 2020-01-08

**Authors:** Yuan Gu, Ximeng Sun, Jingjing Huang, Bin Zhan, Xinping Zhu

**Affiliations:** ^1^Department of Medical Microbiology and Parasitology, School of Basic Medical Sciences, Capital Medical University, Beijing 100069, China; ^2^Department of Pediatrics, Section of Tropical Medicine, Baylor College of Medicine, Houston, Texas 77030, USA

## Abstract

Multiepitope peptide vaccine has some advantages over traditional recombinant protein vaccine due to its easy and fast production and possible inclusion of multiple protective epitopes of pathogens. However, it is usually poorly immunogenic and needs to conjugate to a large carrier protein. Peptides conjugated to a central lysine core to form multiple antigen peptides (MAPs) will increase the immunogenicity of peptide vaccine. In this study, we constructed a MAP consisting of CD4^+^ T cell and B cell epitopes of paramyosin (Pmy) of *Trichinella spiralis* (*Ts*-Pmy), which has been proved to be a good vaccine candidate in our previous work. The immunogenicity and induced protective immunity of MAP against *Trichinella spiralis* (*T. spiralis*) infection were evaluated in mice. We demonstrated that mice immunized with MAP containing CD4^+^ T cell and B cell epitopes (MAP-TB) induced significantly higher protection against the challenge of *T. spiralis* larvae (35.5% muscle larva reduction) compared to the MAP containing B cell epitope alone (MAP-B) with a 12.4% muscle larva reduction. The better protection induced by immunization of MAP-TB was correlated with boosted antibody titers (both IgG1 and IgG2a) and mixed Th1/Th2 cytokine production secreted by the splenocytes of immunized mice. Further flow cytometry analysis of lymphocytes in spleens and draining lymph nodes demonstrated that mice immunized with MAP-TB specifically enhanced the generation of T follicular helper (Tfh) cells and germinal center (GC) B cells, while inhibiting follicular regulatory CD4^+^ T (Tfr) cells and regulatory T (Treg) cells. Immunofluorescence staining of spleen sections also confirmed that MAP-TB vaccination enhanced the formation of GCs. Our results suggest that CD4^+^ T cell epitope of *Ts*-Pmy is crucial in vaccine component for inducing better protection against *T. spiralis* infection.

## 1. Introduction

Trichinellosis is a worldwide food-borne zoonosis spread between animals and people and mainly caused by the infection of *Trichinella spiralis* [1]. People are infected through eating raw or undercooked meat containing infective larvae, mostly from pigs or wild boars [2]. In China, the contaminated pork remains the predominant source of trichinellosis in humans. From 2005 to 2009, 15 outbreaks of human trichinellosis, with 1387 cases and 4 deaths, were reported in three provinces or autonomous regions of Southwestern China; 12 of them (85.71%) were caused by eating the raw or undercooked pork [3]. A pork survey reported that the overall prevalence of *T. spiralis* infection in pigs was 0.61% (5/823) in Henan Province of China, in which 0.91% (5/550) of pigs were infected in Nanyang city alone [4]. It has been estimated that more than 40 million people are at risk of *Trichinella* infection in China [5]. In industrialized countries, although commercially produced pork under controlled management now accounts for about half of the world's pork production, the demand for free-range pork by consumers, especially in Europe and North America, is increasing. In Eastern Europe and Argentina, where traditional free-range backyard-raised pigs are often involved with the feeding of food waste, the infected domestic pork is still blamed on many outbreaks of trichinellosis [6]. Because of the varying degrees of outdoor exposure in free-range systems, there is concern that such exposure will increase the risk of spreading *T. spiralis* from wild animal reservoirs to human beings [6]. It has been reported that rats living on pig farms play an important role in maintaining or spreading this parasite to other animals [2]. Thus, interrupting parasite transmission via vaccination of livestock with a potent and effective vaccine is a practical approach to prevent human trichinellosis.

In the past 30 years, many efforts have been dedicated to develop vaccine against *T. spiralis* infection with the purpose of reducing worm fecundity or decreasing muscle larval and adult worm burdens [1]. The vaccine candidates include excretory-secretory (ES) antigens [7], recombinant proteins [8, 9], and DNA vaccines [10], inducing different levels of partial protective immunity in animal models. However, as a tissue-dwelling helminth, it is difficult to develop an effective vaccine which induces sterile immunity because *T. spiralis* has a complex life cycle, diverse stage-specific antigens, and immune-evasion strategies [11, 12]. Subunit peptide vaccine based on multiple protective epitopes may overcome these problems and thus provides a novel approach to develop vaccines against infectious diseases such as trichinellosis [13].

In our previous study, a promising vaccine candidate, paramyosin (Pmy) of *T. spiralis* (*Ts*-Pmy), has exhibited partially protective immunity against *T. spiralis* infection in mice [14]. A protective B epitope of *Ts*-Pmy, YX1, has been characterized by screening a phage display peptide library with a protective monoclonal antibody (mAb) named as 7E2 [15]. In order to enhance host humoral immunity which plays an important role in the expulsion of *T. spiralis* [16], two potent CD4^+^ T cell epitopes of *Ts*-Pmy identified previously [17] were fused to the protective B epitope of *Ts*-Pmy (YX1) to construct a multiple antigen peptide (MAP-TB) in this study. We have observed that mice immunized with this fused MAP (MAP-TB) based on T/B epitopes of *Ts*-Pmy induced potent protection against *T. spiralis* infection which is associated with enhanced humoral immune responses.

## 2. Materials and Methods

### 2.1. Ethics Statement

This study was performed in accordance with the National Institutes of Health Guidelines for the Care and Use of Experimental Animals. All animal experimental procedures were reviewed and approved by the Institutional Animal Care and Use Committee (IACUC) of Capital Medical University (approval number: AEEI-2015-149).

### 2.2. Mice and Parasites

Six- to eight-week-old female BALB/c (H-2^d^) mice were obtained from the Laboratory Animal Services Center of Capital Medical University (Beijing, China) and raised under specific pathogen-free standard conditions. Each experimental group consisted of ten mice. *T. spiralis* (ISS 533) strain used in this study was maintained in female ICR mice, and the muscle larvae were recovered from the muscle of infected mice using a modified pepsin-hydrochloric acid digestion method as described by Gamble et al. [18].

### 2.3. Synthesis of MAP

**Figure 1:**
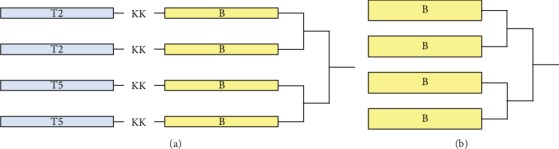
Schematic representation of the construction of MAP-TB (a) and MAP-B (b) used for immunization. In MAP-TB, B cell epitope is colinearly synthesized to T epitope, with a bi-lysine (KK) as a spacer.

MAP-TB and MAP-B constructed in this study are four-branched MAPs containing either two CD4^+^ T cell epitopes (T2 and T5) fused with a B cell epitope (MAP-TB) or only B cell epitope (MAP-B). The T-B peptides are linked at their C terminus to the lysine core of the MAP. Two different CD4^+^ T cell epitopes, T2 and T5, representing the CD4^+^ T cell epitopes P2 and P5 identified in our previous studies [17, 19], were chosen for constructing the MAP-TB (Table 1, Figures 1(a) and 1(b)). B represents the B cell epitope originally designated as YX1, recognized by a protective mAb which conferred partial protection against *T. spiralis* infection by passive transfer [15]. The MAPs were synthesized by Aviva Systems Biology Corporation (China). The synthesis of MAPs utilized a solid-phase synthesis using 9-fluorenylmethoxycarbonyl (FMOC) as a protecting group. The synthesized MAPs were purified by high-performance liquid chromatography (HPLC), then lyophilized and stored desiccated at -80°C before use. The MAPs were also identified by MASS spectrometry, and the purity of MAPs was over 90%. The lyophilized MAPs were dissolved in PBS at 5 mg/ml as stock solutions which were stored at –40°C until use. The working concentration was 0.6 mg/ml in PBS.

### 2.4. Immunization Regimen

**Figure 2:**
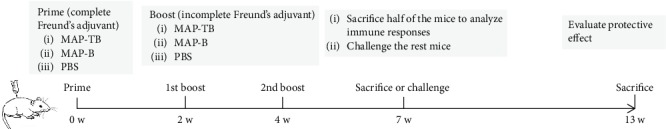
Schematic representation of experimental scheme. Three groups of mice (MAP-TB, MAP-B, and PBS) were immunized three times with Freund's adjuvant and challenged with 400 infective ML of *T. spiralis* to evaluate the protection induced by the MAPs.

To evaluate the immune response induced by the MAP-TB and MAP-B, ten female BALB/c mice were subcutaneously immunized on the back. The MAPs were emulsified with Freund's adjuvant (Sigma-Aldrich, Germany) using complete adjuvant for the initial dose and incomplete adjuvant for the following two booster inoculations at an interval of two weeks. 30 *μ*g of MAP-TB or MAP-B (50 *μ*l) was emulsified with the same volume of adjuvant for a single dose. Another 10 mice were subcutaneously injected with 50 *μ*l PBS emulsified with the same volume of the corresponding adjuvant as the control. One week after the final boost, five mice from each group were sacrificed for collecting sera, spleens, and inguinal lymph nodes (ILNs) to evaluate the induced immune responses. The rest 5 mice were challenged with 400 infective muscle larvae (ML) of *T. spiralis* for evaluating the protection as described below. A representation of experimental design is shown in Figure 2.

### 2.5. Antibody Responses

Anti*-*B cell epitope-specific antibodies induced by MAP immunization were measured by ELISA. Briefly, the plate was coated with 10 *μ*g/well of B cell epitope peptide YX1 in coating buffer overnight at 4°C. After blocking, serial dilutions of sera (0 w, 2 w, 4 w, 7 w, and 13 w) were added at 37°C for 30 min followed with the HRP-conjugated goat anti-mouse IgG (1 : 1000 dilution). The titers of each group were expressed as the geometric mean of the dilution. For IgG subclass detection, the sera were diluted at 1 : 2000 and 1 : 5000, then incubated with Biotin-conjugated Rat Anti-Mouse IgG1 or IgG2a (BD Biosciences, USA) followed by Streptavidin-HRP (BD Biosciences, USA). The color was developed with tetramethylbenzidine substrate (TMB, BD Biosciences, USA) and read at 450 nm.

### 2.6. Cytokine Assay

One week after the final immunization, mice were sacrificed and splenocytes were separated aseptically using mouse lymphocyte separation medium (Dakewe Biotech, China). After being centrifuged, the spleen cells were resuspended and adjusted to 1 × 10^7^ cells/ml in complete RPMI-1640 supplemented with 10% FBS, penicillin (100 U/ml), and streptomycin (100 *μ*g/ml). For *in vitro* stimulation, a total of 1 × 10^6^ splenocytes were incubated with mixed peptides (2.5 *μ*g/ml T2 and T5 individually) in 200 *μ*l of complete RPMI-1640 for 48 h at 37°C in a humidified atmosphere containing 5% CO_2_. Splenocytes stimulated simultaneously with ConA (Sigma-Aldrich, USA; 5 *μ*g/ml) were served as positive controls. The cytokines IFN-*γ*, IL-2, IL-4, IL-5, and IL-6 were detected using the corresponding ELISA kit (BioLegend, USA), according to the manufacturer's instructions.

### 2.7. Flow Cytometric Analysis

Spleens and ILNs of mice were harvested on day 7 after the final immunization. The lymphocytes were collected and analyzed by flow cytometry. The frequencies of CXCR5^+^PD-1^+^CD3^+^CD4^+^ Tfh cells (in spleens), CXCR5^+^PD-1^high^CD3^+^CD4^+^ Tfh cells (in ILNs), and CD25^+^FoxP3^+^CD3^+^CD4^+^ Treg cells (in spleens) presented within the total CD4^+^ T cells; the frequencies of GL7^+^Fas^+^B220^+^ germinal center (GC) B cells presented within the total B220^+^ B cells; and the frequencies of CXCR5^+^FoxP3^+^CD3^+^CD4^+^ Tfr cells presented within the CD4^+^ T cells (in spleens and ILNs) were detected. Dead cells were excluded by viability dye staining, and duplicates were excluded by FSC/A and FSC/W gating analysis. Cells were analyzed by a BD LSRFortessa™ Flow Cytometry (BD Biosciences, USA). Data were acquired and analyzed by BD FACSDIVA™ version 8.0.2 (BD Biosciences, USA).

For surface staining, splenocytes were blocked with anti-CD16/CD32 mAb (Clone: 93, BD Pharmingen™, USA) and stained with the following antibodies: anti-CD3e-Percp-Cyanine5.5 (Clone: 145-2C11, eBioscience, USA), anti-CD4-FITC (Clone: RM4-5, eBioscience, USA), anti-PD-1-PE (Clone: J43, eBioscience, USA), and anti-CXCR5-APC (Clone: SPRCL5, eBioscience, USA) for Tfh cell analysis; with anti-GL7-FITC (Clone: GL7, BD Pharmingen™, USA), anti-Fas-PE (Clone: Jo2, BD Pharmingen™, USA), and anti-B220-PerCP-Cy5.5 (Clone: RA3-6B2, BD Pharmingen™, USA) mAb for GC B cell analysis. For viability dye staining, Fixable Viability Dye eFluor™ 450 (eBioscience, USA) was added with surface staining antibodies.

For intracellularly staining (Treg/Tfr), the splenocytes were blocked with anti-CD16/CD32 mAb (Clone: 93, BD Pharmingen™, USA), stained with anti-CD3e-Percp-Cyanine5.5, anti-CD4-FITC and anti-CXCR5-APC, or anti-CD25-APC (Clone: PC61.5, eBioscience, USA), then fixed with FoxP3 Fixation/Permeabilization working solution (eBioscience, USA) and permeabilized with Permeabilization Buffer (eBioscience, USA). After that, the cells were intracellularly stained with anti-FoxP3-PE (Clone: FJK-16s, eBioscience, USA).

### 2.8. Immunofluorescence Assay

The spleens from BALB/c mice were separated one week after the final immunization and embedded in Optimal Cutting Temperature (OCT) Compound (SAKURA, USA). The tissues were frozen at -80°C before sectioning (8 *μ*m) on a Cryostat (Leica, Germany). After being fixed in cold acetone and blocked with 1% BSA in PBS at room temperature for 1 h, the sections were incubated with Biotinylated Peanut Agglutinin (PNA, 1 : 100 dilution, VECTOR, USA) at 4°C overnight. DyLight™ 488 Streptavidin (1 : 100 dilution, BioLegend, USA) was used as the secondary antibody at room temperature for 1 h. At last, Alexa Fluor® 647-conjugated anti-mouse CD45R (1 : 150 dilution, Clone RA3-6B2, BioLegend, USA) was incubated at room temperature for 1 h. The sections were scanned under a Pannoramic SCAN instrument (3DHISTECH, Hungary). For quantification the area of GCs, spleen sections of three mice from each group were analyzed by ImageJ software.

### 2.9. Challenge Experiment

One week after the third immunization, the remaining five mice of each group were challenged orally with 400 *T. spiralis-*infective ML. Six weeks after the infection, the larvae from the muscle of each infected mouse were collected and counted. The reduction rate of ML burden was calculated based on the recovered larvae per gram (LPG) muscle from the mice immunized with MAP-TB or MAP-B versus those from the PBS control group. Specifically, it was calculated as follows: worm burden reduction rate (%) = (1 − mean number of LPG in vaccinated mice/mean number of LPG in control mice) × 100%. Three independent experiments were carried out.

### 2.10. Statistical Analysis

Statistical analyses were performed with One-way ANOVA using SPSS for Windows, version 17.0. All data are expressed as the mean value + standard deviations (SD) or the mean value ± SD. *P* value < 0.05 were considered statistically significant.

## 3. Results

### 3.1. Mice Immunized with MAP-TB Vaccine Produced Significant Higher Antibody Responses

**Figure 3:**
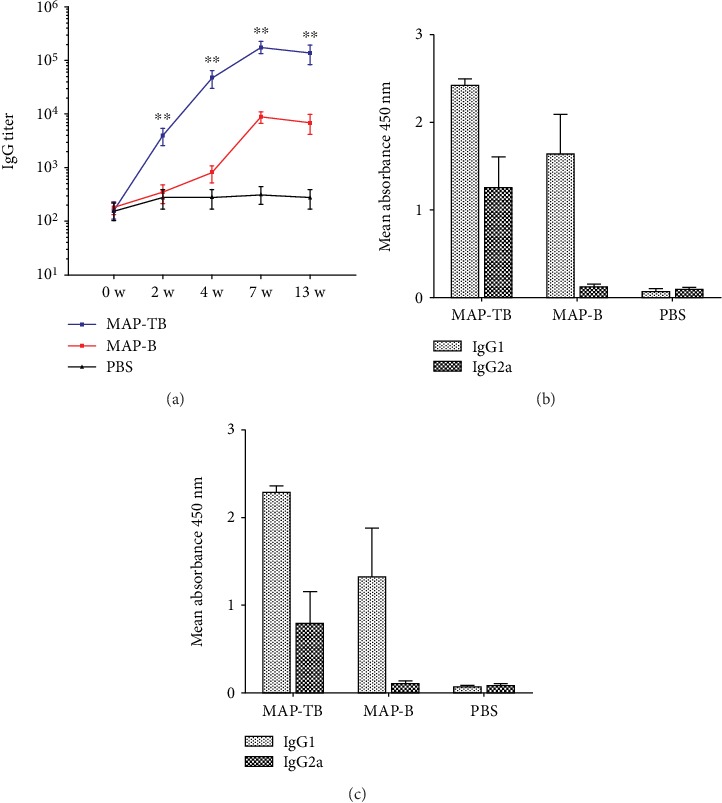
Serological antibody responses induced by immunization of MAP-TB or MAP-B measured by ELISA. (a) Specific IgG titers against B cell epitope peptide YX1 in the sera of mice immunized with MAP-TB or MAP-B on 0 w, 2 w, 4 w, 7 w, and 13 w. The total IgG is shown as the geometric mean titer of five mice within the group. ∗∗ indicates that anti-YX1 IgG titers in five mice immunized with MAP-TB were greatly elevated after the first immunization and were significantly higher than those in mice immunized with MAP-B or PBS (*P* < 0.01). (b, c) The OD_450_ of subtype IgG1 and IgG2a responses in the sera of mice immunized with MAP-TB, MAP-B, or PBS at a dilution of 1 : 2,000 (b) and 1 : 5,000 (c). The values are shown as the mean + SD. The data was shown as one representative experiment out of three.

BALB/c mice were immunized with MAP-TB and MAP-B for three times, and the sera were collected on 0 w, 2 w, 4 w, 7 w, and 13 w. B cell epitope peptide YX1 was used as a coating antigen, and the antibody titers against YX1 were measured by ELISA. Anti-YX1 IgG titers in five mice immunized with MAP-TB were greatly elevated after the first immunization and significantly higher than those in mice immunized with MAP-B or PBS (Figure 3(a), *P* < 0.01). The IgG antibody subclass was determined at a 1 : 2000 dilution (Figure 3(b)) and 1 : 5000 (Figure 3(c)) and revealed that MAP-TB induced both IgG1 and IgG2a responses, with IgG1 being predominant. However, MAP-B contains only B epitope and immunization of MAP-B induced a Th2 polarization direction which showed mainly IgG1 and impaired production of IgG2a.

### 3.2. MAP-TB Vaccination Induced a Mixed Th1 and Th2 Cytokine Response in Mice

**Figure 4:**
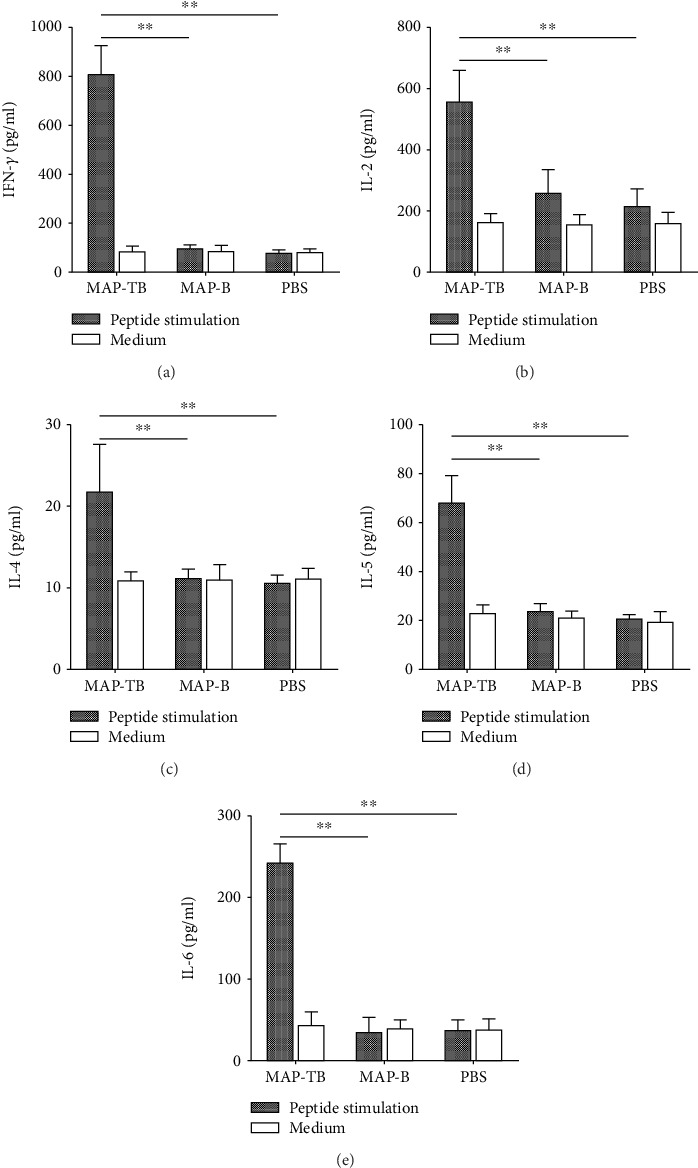
Cytokines secreted by splenocytes from immunized mice upon stimulation of mixed peptides T2 and T5 *in vitro*. Splenocytes secreted IFN-*γ* (a), IL-2 (b), IL-4 (c), IL-5 (d), and IL-6 (e) were detected by ELISA seven days after the final immunization. Splenocytes of each sample were stimulated simultaneously with ConA (5 *μ*g/ml) as positive controls. All cytokines were greatly elevated upon stimulation with ConA, and the highest levels were observed as follows: IFN-*γ*—1280 pg/ml; IL-2—640 pg/ml; IL-4—60 pg/ml; IL-5—125 pg/ml; and IL-6—857 pg/ml (data not shown in the figure). The results are shown as the mean + SD (one representative experiment out of three). ^∗∗^*P* < 0.01 (*n* = 5).

The cytokines IFN-*γ*, IL-2, IL-4, IL-5, and IL-6 secreted by splenocytes of immunized mice upon stimulation of mixed peptides T2 and T5 *in vitro* were detected by corresponding specific ELISA. The levels of the typical Th1 cytokines (IFN-*γ* and IL-2), Th2 cytokines (IL-4 and IL-5), and IL-6 in the culture supernatants of splenocytes upon stimulation were significantly elevated in mice immunized with MAP-TB as compared with mice immunized with MAP-B or PBS (Figure 4). However, there was no significant elevation of all cytokine responses in mice immunized with MAP-B compared with the PBS control group. These results indicated that MAP-TB vaccination induced a mixed Th1 and Th2 cytokine response in mice.

### 3.3. MAP-TB Vaccination Enhanced Expansion of Tfh Cells and Germinal Center B Cells

**Figure 5:**
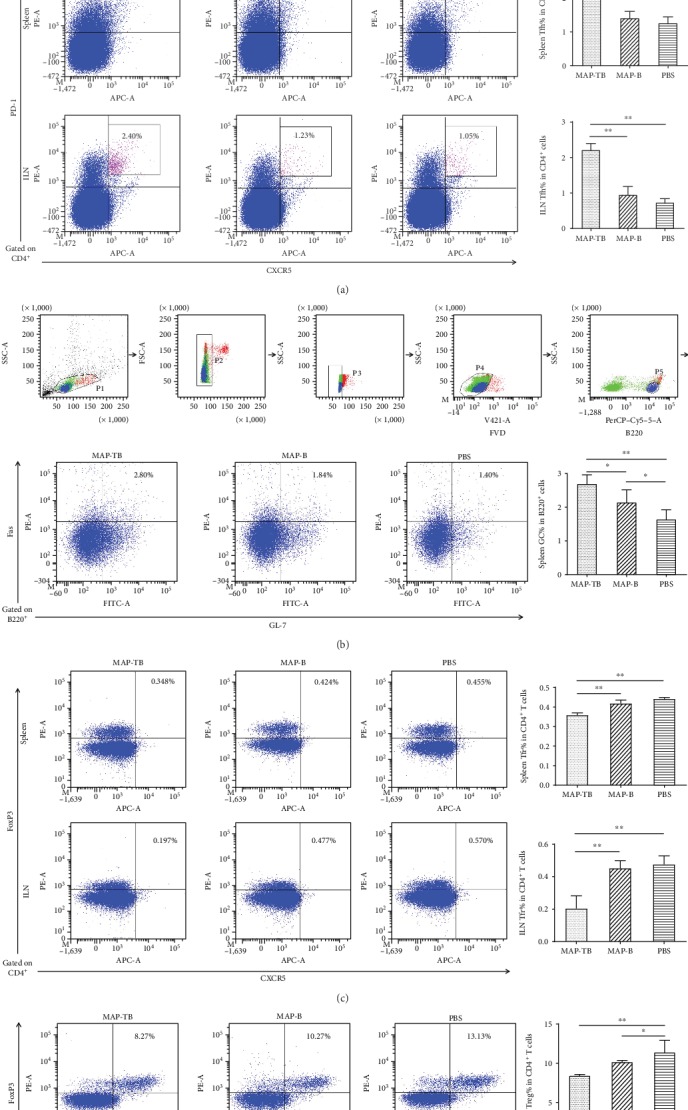
Tfh cells, GC B cells, Tfr cells, and Treg cells in the splenocytes and ILN lymphocytes of immunized mice were determined by flow cytometric analysis seven days after the final immunization. MAP-TB vaccination enhanced expansion of Tfh cells and GC B cells, while decreasing the frequencies of Tfr cells and Treg cells compared with the MAP-B or PBS group. For Tfh, Tfr, and Treg analysis, CD3^+^CD4^+^ T cells were gated and the gating strategies are shown in (a). (a) Representative dot plots of Tfh cells gated on CD4^+^ T cells of spleens and ILNs. Percentages in the upper right quadrant indicated the frequencies of CXCR5^+^PD-1^+^CD3^+^CD4^+^ Tfh cells within total CD4^+^ T cells. (b) Representative dot plots of GC B cells gated on B220^+^ B cells of the spleens. Percentages in the upper right quadrant indicated the frequencies of GC B cells within total B220^+^ B cells. (c) Representative dot plots of Tfr cells gated on CD4^+^ T cells of the spleens and ILNs. Percentages in the upper right quadrant indicated the frequencies of CXCR5^+^FoxP3^+^CD3^+^CD4^+^ cells within the CD4^+^ T cells of the spleens and ILNs. (d) Representative dot plots of Treg cells gated on CD4^+^ T cells of the spleens. Percentages in the upper right quadrant indicated the frequencies of CD25^+^FoxP3^+^CD3^+^CD4^+^ cells within the total CD4^+^ T cells of the spleens. The graphs are shown as the mean + SD (one representative experiment out of three). ^∗∗^*P* < 0.01; ^∗^*P* < 0.05 (*n* = 5).

The production of high-affinity antibodies relies on the complex interaction of B cells with Tfh cells in the GC reaction. Seven days after the final immunization, lymphocytes of spleens and ILNs were harvested and analyzed by flow cytometry to determine the frequencies of CXCR5^+^PD-1^+^CD3^+^CD4^+^ Tfh cells (in spleens), CXCR5^+^PD-1^high^CD3^+^CD4^+^ Tfh cells (in ILNs), GL7^+^Fas^+^B220^+^ GC B cells (in spleens), CXCR5^+^FoxP3^+^CD3^+^CD4^+^ Tfr cells [20] (in spleens and ILNs), and CD25^+^FoxP3^+^CD3^+^CD4^+^ Treg cells (in spleens). Representative dot plots of flow cytometric analysis are shown in Figure 5. It has been proved that CXCR5^+^PD-1^high^CD3^+^CD4^+^ T cells residing in lymph node GC represent a specific Tfh subset that correlate with B cell maturation and IgG production [21]. The frequencies of Tfh cells within total CD4^+^ T cells of spleens and ILNs and GC B cells within total B220^+^ B cells of spleens increased in mice immunized with MAP-TB compared with the MAP-B or PBS group (Figures 5(a) and 5(b)). Consistently, the frequencies of Tfr cells within CD4^+^ T cells of spleens and ILNs and the frequencies of Treg cells within the CD4^+^ T cells of spleens decreased significantly in mice immunized with MAP-TB compared with the other two groups (Figures 5(c) and 5(d)).

**Figure 6:**
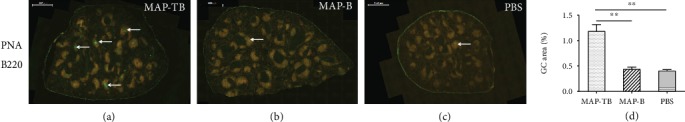
MAP-TB vaccination enhanced the formation of GCs in the spleen sections of immunized mice stained by PNA (green) and B220 (orange). The scale bar represents 1000 *μ*m. The areas of GCs were normalized by section area, and spleen sections of three mice from each group were analyzed by ImageJ software (d).

The formation of GCs through vaccination could be confirmed by PNA staining [22]. MAP-TB vaccination greatly enhanced the formation of GCs in the spleens of mice compared to the MAP-B or PBS group confirmed by immunofluorescence staining (Figure 6).

### 3.4. Partially Protective Immunity Elicited by MAP-TB Immunization

Challenge experiments demonstrated that mice immunized with MAP-TB induced 35.5% ML reduction compared to the PBS control group (*P* < 0.01), which was also significantly higher than MAP-B immunization (12.4% ML reduction, *P* < 0.05). However, there was no significant difference in the ML burden between the MAP-B and PBS groups (Table 2). These results indicated that MAP-TB immunization elicited partial protection against *T. spiralis* infection, whereas MAP-B immunization did not induce significant protection (*P* < 0.01).

## 4. Discussion

During the past decades, researchers have been devoted to developing vaccines against trichinellosis utilizing different vaccine platforms, such as crude worm antigens, recombinant proteins, and DNA vaccines. As *T. spiralis* cannot be cultured *in vitro*, it is difficult to obtain a large amount of parasite antigens. In recent years, researchers have focused on recombinant proteins and DNA vaccines. For example, recombinant fructose-1,6-bisphosphate aldolase [23], serine protease [24], DNase II enzyme [25], and enolase [26] from *T. spiralis* exhibited muscle larva reductions range from 17.7% to 62.1%. Different DNA vaccines showed muscle larva reductions from 15.8% to 71.84% after *T. spiralis* larval challenge [10, 26, 27]. A few researches have been done on peptide vaccines against trichinellosis. It was first reported in 1995 that a 40-mer synthetic peptide vaccine induced a 64.3% adult worm reduction in subcutaneously immunized mice [28]. Seven years later, the same research group reported that intranasal administration of a 30-mer peptide antigen with cholera toxin B female significantly reduced worm fecundity (33.3%) compared to the controls [29]. Another research group reported that immunization of mice with an attenuated *Salmonella* strain displaying a 30-mer peptide using the ShdA autotransporter induced significantly adult worm reduction (61.83%) against *T. spiralis* infection [30]. All of the above mentioned peptides are from the *T. spiralis* gp43 antigen, which has been proved to be a highly immunodominant protein.

In our previous study, *Ts*-Pmy has been proved to be a good vaccine candidate antigen which induced significant protection in immunized mice against the challenge of *T. spiralis-*infective larvae [14]. However, as a big protein consisting of 885 amino acids with a predicted molecular mass of 102 kDa, it is difficult to be expressed as a soluble recombinant protein in a prokaryotic expression system. Even in the eukaryotic expression system, only a small portion of protein could be expressed as soluble protein, which prevents its scale-up production as a recombinant protein vaccine. In addition to the difficulties in the yield of full-length recombinant protein, the complexity of the whole protein antigen may cause undesired detrimental side effects [31]. In recent years, peptide production becomes easily reproducible, fast, and cost-effective due to the advances in the peptide synthesis. In addition, chemical synthesis could remove the concerns associated with the biological contamination of the expression system antigens in the recombinant protein production. Peptide vaccines also have some other advantages, such as being water soluble and having high stability under simple storage conditions (generally does not require “cold chain”) [32]. Due to these advantages, peptide-based vaccines are now playing an important role in the development of cancer and infective disease vaccine [13, 33]. Some clinical trials for peptide-based vaccines have been successfully tested as potential candidates for cancer therapeutic in recent years [33].

However, peptide vaccine also has some disadvantages, such as poor immunogenicity and the need to conjugate to a large carrier protein (e.g., KLH and BSA). To increase the immunogenicity of a peptide-based vaccine, a new strategy of multiple antigenic peptides (MAPs) becomes a popular alternative. MAPs are peptides that are artificially branched by utilizing a lysine-based central backbone to which multiple peptide chains could be conjugated. The branched peptides sometimes can greatly increase their immunological responses because of the high molar ratio of peptide antigen to the core molecule and the high molecular weight which make them more immunogenic without the need of conjugation to a carrier protein [32, 34–36]. MAP vaccines have been studied in several infectious diseases, such as lymphatic filariasis [37], *Plasmodium falciparum* (*P. falciparum*) [38], *Yersinia pestis* [39], and HIV [40]. Clinical studies have been carried out in MAP vaccine against *P. falciparum* sporozoites [41]. In lymphatic filariasis, thioredoxin-transglutaminase MAP conferred a significantly higher protection of 63.04% than the whole protein cocktail vaccine did (55.8%) in jird models [37]. In *P. falciparum*, anti-MAP-1 (circumsporozoite protein-based) antibodies blocked the invasion of HepG2 liver cells by *P. falciparum* sporozoites (highest, 95.16% in HLA-A2 C57BL/6; lowest, 11.21% in BALB/c) [38]. This study revealed that the immune response induced by MAP was generally MHC dependent. It highlights the prospect of MAP constructs that may generate highly effective antimalarial responses in populations of genetically diverse HLA types.

Among peptide vaccines, epitope-composed one has some other unique advantages and becomes a good choice for vaccine development. This type of vaccine allows us to focus on the epitopes that possess strong immunogenicity and could induce robust protective immune effects in immunized animals. Identification and correct selection of these epitopes are a crucial step in the design of an epitope-based peptide vaccine. The immunogenic epitopes on the protein of interest should be identified either by epitope prediction algorithms using bioinformatics tool [42] or by screening with antibodies [43]. These epitopes should be tested and confirmed for their ability to induce strong, long-lasting humoral (B cell or Th2 epitopes) and/or cellular immunity (CTL or Th1 epitopes) against the target pathogen [32, 42]. In this study, we constructed a MAP based on T/B cell epitope of *Ts*-Pmy and evaluated its immunogenicity and the induced protective immunity compared to the B cell epitope-conjugated MAP.

Previous studies showed that Th2 immune response was important in protective immunity against *T. spiralis* infection [16]. Therefore, Th2 epitopes should be an essential part of a vaccine against *T. spiralis* infection. During the initiation of immune response, the major histocompatibility complex- (MHC-) II combined with processed antigen epitope in antigen presenting cells trigger the activation of T helper cells which further activate cellular immunity and/or humoral immunity. In our previous work, based on the BALB/c mouse model, H-2^d^-restricted CD4^+^ T cell epitopes (I-A^d^ and I-E^d^) of *Ts*-Pmy were predicted using the SYFPEITHI database. The epitope peptides could stimulate splenocytes of r*Ts*-Pmy-immunized mice to secrete the Th2 cytokines IL-4 and IL-5. It has been further verified that these epitopes were immunodominant Th2 epitopes of *Ts*-Pmy by experiments *in vitro* and *in vivo* [17]. In the experiments of identifying and characterizing of CD4^+^ T cell epitopes, stimulation of splenocytes from mice immunized with r*Ts*-Pmy produced the highest IL-5 by peptide T5, while T2 stimulated the highest production of Th2 cytokine IL-4, as compared to the other T cell epitopes. T5 also stimulated splenocytes of mice immunized with T5 to produce the highest IL-4 among all the candidate CD4^+^ T cell epitopes [17]. According to these results, T5 and T2 were selected as the CD4^+^ T cell epitopes to construct MAP in this study. In addition, a protective B epitope of *Ts*-Pmy, YX1, located between 88 and 107 amino acids of *Ts*-Pmy, has been identified by the recognition of a mAb 7E2 which passively transferred the protection against *T. spiralis* infection in naïve mice [15]. To evaluate whether Th2 epitopes could coordinate B cell epitope to produce the better protection against *T. spiralis* infection compared with B cell epitope alone, two types of MAP including MAP-TB and MAP-B were constructed. MAP-TB combines two Th2 epitopes and one B cell epitope while MAP-B contains only B cell epitope (Figure 1). The induced immune responses and protective effects against *T. spiralis* infection by immunization with these two MAP vaccines were further evaluated and compared.

Our previous study showed that a recombinant multiepitope protein (rMEP) combined four CD4^+^ T cell epitopes and one B cell epitope produced a 55.4% of muscle larval reduction [19]. In this study, we demonstrated that mice immunized with MAP-TB induced significantly higher protection against the challenge of *T. spiralis*-infective larvae (35.5% ML reduction) compared to the MAP-B which induced only 12.4% ML reduction. MAP-TB did not induce comparable protection as that induced by rMEP possibly due to the less protective epitopes included in the construct of MAP-TB. Due to technical limitations of MAP synthesis, only two of four CD4^+^ T cell epitopes and one B cell epitope were constructed in MAPs in this study. The nonsterilizing immunity or low protection is a dilemma not only for vaccine development against Trichinella infection but also for all other helminth infections. For example, one of the most well-researched helminth diseases, schistosomiasis, the bar to achieve protective efficacy in humans was set at a consistent induction of 40% protection or better by the World Health Organization (WHO), and although this is a modest goal, it is yet to be reached with the six most promising schistosomiasis vaccine candidates (Sm28GST, IrV5, Sm14, paramyosin, TPI, and Sm23) [44, 45]. Vaccine against hookworm with less than 30% reduction in worm burden was also tested in clinical trials [46, 47]. The low protection induced by single vaccine immunization for helminth infection may be caused by the complexity of the life cycle, diversity of stage-specific antigens, immune-evasion strategies, and the modulatory effect of host responses [48]. Indeed, the pathology of parasites is directly related to the number of worms harbored by the host. Instead, the major benefit of a vaccine would be the reduction in worm burden with a concomitant reduction in morbidity, especially for the helminthic parasites, which induce nonsterilizing immunity in the host [49].

In this study, MAP-TB induced robust humoral immune response with higher IgG titers than MAP-B did. IgG subtype examination showed that MAP-TB immunization induced both IgG1 and IgG2a (with IgG1 predominant), while MAP-B mainly induced IgG1, indicating MAP-TB stimulated both Th1 and Th2 responses. The cytokine profiles secreted by the splenocytes also showed that MAP-TB immunization induced not only Th2 cytokines (IL-4 and IL-5) but also Th1 cytokines (IFN-*γ* and IL-2). Th1 cytokine response was also elevated, and it was speculated that the epitopes were short peptides and they might be cross-presented by the dendritic cells.

Further flow cytometry analysis of the lymphocytes in the spleens and draining lymph nodes demonstrated that mice immunized with MAP-TB specifically enhanced the generation of Tfh cells and GC B cells and inhibited Tfr/Treg cells. Immunofluorescence staining of spleen sections also confirmed that MAP-TB vaccination enhanced the formation of GCs. All results indicated that MAP containing Th2 and B cell epitopes of *Ts*-Pmy induced better protection than MAP with B cell epitope alone, which associates with enhanced humoral immune responses, augmentation of Tfh and GC B cells, and inhibition of Tfr and Treg cells. It also indicated that B cell epitope alone could not induce strong humoral immune response, which is associated with decreased generation of Tfh and GC B cells.

Tfh cells are the unique CD4^+^ T follicular helper cells that provide cognate help to B cells to induce high-affinity antibody production in GCs [50]. Tfh cells depend on CXCR5 to localize in the follicular regions of lymphoid organs and maintain stable contact with antigen-primed B cells [51]. GCs support intense B cell clonal expansion, somatic hypermutation, selection of high-affinity B cells, and class switching of immunoglobulin genes. The products of the GC reaction are memory B cells and long-lived plasma cells that secrete high-affinity antibodies [22]. Mounting evidence suggests a strong correlation between the frequencies of Tfh cells and antigen-specific antibody responses, and more importantly, it was further proved in the human immune system [52]. Except for the inducement of Tfh proliferation, MAP-TB immunization also induced an elevation of IL-6 which has been shown to be an important regulator for the differentiation of Tfh cells [53]. It was reported that IL-6 played a pivotal role in shaping the acquired immune response by promoting the differentiation of B cells into immunoglobulin producing plasma cells and increasing the gamma globulin level in serum [54]. In addition, IL-6 is also an essential cytokine that transmits defense signals from a pathogen invasion or tissue damage site to stimulate acute phase reactions, immune responses, hematopoiesis, and various internal organs to prepare for host defense [55].

As a strong association of Tfh cells with multiple systemic and mucosal antibody responses was testified in many studies, many explorations of vaccine strategies have been made to enhance the generation of Tfh cells through the modulation of vaccine regimens. For example, an oil-in-water adjuvant, MF59, has been shown to promote GC B cell differentiation and Tfh induction [56]. Other vaccine strategies, such as incorporating DNA priming and protein boosting [57] or nanoparticle vaccines [58], have showed to expand Tfh cell populations and promote GC development, leading to enhanced humoral immunity. Antigen dose also has a positive impact on the induced frequencies of Tfh cells and subsequent serologic responses [59]. Given the importance of innate immune signals in shaping adaptive immune responses [60], a particular innate immune pathway with Tfh cell-skewing ability, such as TLR8, has been identified for the rational design of Tfh cell-targeted vaccine adjuvants [61]. In our future studies, efforts will be targeted to trigger stronger antigen-specific Tfh responses induced by the peptide vaccines.

T follicular regulatory (Tfr) cells are a specialized subset of effector Treg cells that inhibit antibody production [62]. Successful humoral immunity is a delicate balance between stimulatory Tfh cells and inhibitory Tfr cells and not simply a result of the total number of Tfh cells [63]. Tfh and Tfr cells tightly control the size and output of the GC reaction and thus make them key targets to manipulate the vaccine design. Increasing Tfh cell formation and/or function or reducing the suppression exerted by Tfr cells in GC may be a rational strategy to improve vaccine response and efficacy [64]. Our study showed the increased frequencies of Tfh and the decreased frequencies of Tfr in the spleens and ILNs correlated with the expansion of antibody responses, which is consistent with previous studies [63, 65].

Treg cells are considered negative regulators of immune response which could suppress the activation, proliferation, and effector functions (such as cytokines production) of natural killer (NK) and NKT cells, B cells, CD4^+^ and CD8^+^ T cells, and antigen-presenting cells, *in vitro* and *in vivo* [66]. As a survival strategy of helminthic parasites, chronic helminth infection stimulates Tregs to produce regulatory and anti-inflammatory cytokines that reduce host immune responses to the invading worms [67]. Many helminth infections, such as *Heligmosomoides polygyrus* [68], *Schistosoma japonicum* [69], *Schistosoma mansoni* [70], and *Brugia malayi* [71], are known to stimulate an increased number of Tregs. Recently, our research group has found that Treg cells were significantly induced at the intestinal stage (6 days post infection) and newborn larva migration stage (15 days post infection) during the early *T. spiralis* infection [72]. In our study, we identified that immunization with MAP-TB decreased Treg (*P* = 0.052) and Tfr cells and stimulated Tfh and GC B cells in dLNs and spleens of immunized mice compared to MAP-B alone, indicating the addition of T-epitope in MAP-B could offset the immune inhibition induced by helminth infection and boost humoral immune response to vaccine antigen. This may also partly explain the protective effects induced by the immunization of MAP-TB and MAP-B.

In the current study, we found that peptide vaccine MAP-TB comprising CD4^+^ T cell epitopes could induce better protection against *T. spiralis* infection, which was associated with enhanced humoral immune response, compared to the MAP-B and PBS groups. The results from this study indicate that B-epitope alone is not efficient to induce robust humoral immune response. It is necessary to combine CD4^+^ T cell epitopes to construct an epitope-based peptide vaccine in order to induce better protection against *T. spiralis* infection. Similar studies on infective diseases also showed that potent CD4^+^ T cell epitope is a key vaccine component to elicit robust immune responses [73, 74]. As a multivalent antigen peptide vaccine could not only better cover the diversity of natural pathogen antigen and may even target several life stages of the pathogen but also better match the genetic variability of the host immune system [32], therefore, it is a promising strategy for developing vaccine against multicellular helminth infections such as *T. spiralis*. In our future study, including more protective epitopes (both T and B cell epitopes) in MAP constructs would be a practical way to improve protection.

## Figures and Tables

**Table 1:** Amino acid sequences and positions of CD4^+^ T cell epitopes and B cell epitope of *Ts*-Pmy.

Epitope	Position in *Ts*-Pmy	Amino acid sequence
T2 (P2)	528-542	QFEIDRLAAALADAE
T5 (P5)	610-624	AIAQRKLSALSAELE
B (YX1)	88-107	EEAEGTTDAQIDANRKRESE

**Table 2:** Protection elicited by MAP immunization against challenge with 400 *T. spiralis* ML in mice.

Experiment	MAP-TB	MAP-B	PBS
	(Mean LPG ± SD/ML burden reduction)		
1st	3799 ± 565/35.5%^ab^	5156 ± 543/12.4%	5886 ± 1101/-
2nd	4024 ± 637/37.2%^ab^	5469 ± 701/14.6%	6403 ± 1261/-
3rd	4105 ± 583/33.0%^ab^	5444 ± 620/11.1%	6124 ± 1086/-

Three independent challenge experiments were carried out to evaluate the protection induced by MAP constructs. ^a^*P* < 0.01 compared with PBS control group; ^b^*P* < 0.05 compared with MAP-B group (*n* = 5).

## Data Availability

The data used to support the findings of this study are available within the article, or from the authors upon reasonable request.

## Abbreviations

ES: Excretory-secretory
GC: Germinal center
HPLC: High-performance liquid chromatography
ILN: Inguinal lymph nodes
LPG: Larvae per gram
mAb: Monoclonal antibody
MAP: Multiple antigen peptide
MHC: Major histocompatibility complex
ML: Muscle larvae
NK: Natural killer
OCT: Optimal cutting temperature
Pmy: Paramyosin
PNA: Peanut agglutinin
Tfh cell: T follicular helper cell
Tfr cell: T follicular regulatory cell
TMB: Tetramethylbenzidine
Treg cell: Regulatory T cell
*Ts*-Pmy: Paramyosin of *Trichinella spiralis*

## References

[1] Zhang N., Li W., Fu B. (2018). Vaccines against *Trichinella spiralis*: progress, challenges and future prospects. *Transboundary and Emerging Diseases*.

[2] Bilska-Zajac E., Rozycki M., Antolak E. (2018). Occurrence of *Trichinella* spp. in rats on pig farms. *Annals of Agricultural and Environmental Medicine*.

[3] Cui J., Wang Z. Q. (2011). An epidemiological overview of swine trichinellosis in China. *Veterinary Journal*.

[4] Jiang P., Zhang X., Wang L. A. (2016). Survey of *Trichinella* infection from domestic pigs in the historical endemic areas of Henan province, Central China. *Parasitology Research*.

[5] Bai X., Hu X., Liu X., Tang B., Liu M. (2017). Current research of trichinellosis in China. *Frontiers in Microbiology*.

[6] Murrell K. D. (2016). The dynamics of *Trichinella spiralis* epidemiology: out to pasture?. *Veterinary Parasitology*.

[7] Quan F. S., Matsumoto T., Lee J. B. (2004). Immunization with *Trichinella spiralis* Korean isolate larval excretory-secretory antigen induces protection and lymphocyte subset changes in rats. *Immunological Investigations*.

[8] Bi K., Yang J., Wang L., Gu Y., Zhan B., Zhu X. (2015). Partially protective immunity induced by a 20 kDa protein secreted by *Trichinella spiralis* stichocytes. *PLoS One*.

[9] Song Y. Y., Zhang Y., Ren H. N. (2018). Characterization of a serine protease inhibitor from *Trichinella spiralis* and its participation in larval invasion of host's intestinal epithelial cells. *Parasites & Vectors*.

[10] Li J. F., Guo K. X., Qi X. (2018). Protective immunity against *Trichinella spiralis* in mice elicited by oral vaccination with attenuated *Salmonella*-delivered TsSP1.2 DNA. *Veterinary Research*.

[11] Sun R., Zhao X., Wang Z. (2015). *Trichinella spiralis* paramyosin binds human complement C1q and inhibits classical complement activation. *PLOS Neglected Tropical Diseases*.

[12] Zhao L., Shao S., Chen Y. (2017). *Trichinella spiralis* calreticulin binds human complement C1q as an immune evasion strategy. *Frontiers in Immunology*.

[13] Li Y., Zheng K., Tan Y. (2019). A recombinant multi-epitope peptide vaccine based on MOMP and CPSIT_p6 protein protects against *Chlamydia psittaci* lung infection. *Applied Microbiology and Biotechnology*.

[14] Yang J., Gu Y., Yang Y. (2010). *Trichinella spiralis*: immune response and protective immunity elicited by recombinant paramyosin formulated with different adjuvants. *Experimental Parasitology*.

[15] Wei J., Gu Y., Yang J. (2011). Identification and characterization of protective epitope of *Trichinella spiralis* paramyosin. *Vaccine*.

[16] Angkasekwinai P., Sodthawon W., Jeerawattanawart S., Hansakon A., Pattanapanyasat K., Wang Y. H. (2017). ILC2s activated by IL-25 promote antigen-specific Th2 and Th9 functions that contribute to the control of *Trichinella spiralis* infection. *PLoS One*.

[17] Gu Y., Huang J., Wang X. (2016). Identification and characterization of CD4^+^ T cell epitopes present in *Trichinella spiralis* paramyosin. *Veterinary Parasitology*.

[18] Gamble H. R., Bessonov A. S., Cuperlovic K. (2000). International commission on Trichinellosis: recommendations on methods for the control of *Trichinella* in domestic and wild animals intended for human consumption. *Veterinary Parasitology*.

[19] Gu Y., Sun X., Li B., Huang J., Zhan B., Zhu X. (2017). Vaccination with a paramyosin-based multi-epitope vaccine elicits significant protective immunity against *Trichinella spiralis* infection in mice. *Frontiers in Microbiology*.

[20] Vaeth M., Muller G., Stauss D. (2014). Follicular regulatory T cells control humoral autoimmunity via NFAT2-regulated CXCR5 expression. *The Journal of Experimental Medicine*.

[21] Xu H., Wang X., Lackner A. A., Veazey R. S. (2014). PD-1^HIGH^ follicular CD4 T helper cell subsets residing in lymph node germinal centers correlate with B cell maturation and IgG production in rhesus macaques. *Frontiers in Immunology*.

[22] Victora G. D., Nussenzweig M. C. (2012). Germinal centers. *Annual Review of Immunology*.

[23] Yang Y., Bai X., Li C. (2019). Molecular characterization of fructose-1, 6-bisphosphate aldolase from *Trichinella spiralis* and its potential in inducing immune protection. *Frontiers in Cellular and Infection Microbiology*.

[24] Sun G. G., Lei J. J., Ren H. N. (2019). Intranasal immunization with recombinant *Trichinella spiralis* serine protease elicits protective immunity in BALB/c mice. *Experimental Parasitology*.

[25] Qi X., Yue X., Han Y. (2018). Characterization of two *Trichinella spiralis* adult-specific DNase II and their capacity to induce protective immunity. *Frontiers in Microbiology*.

[26] Zhang X., Xu L., Song X., Li X., Yan R. (2018). Molecular cloning of enolase from *Trichinella spiralis* and the protective immunity in mice. *Acta Parasitologica*.

[27] Wang L., Wang X., Bi K. (2016). Oral vaccination with attenuated *Salmonella typhimurium*-delivered *Ts*Pmy DNA vaccine elicits protective immunity against *Trichinella spiralis* in BALB/c mice. *PLOS Neglected Tropical Diseases*.

[28] Robinson K., Bellaby T., Chan W. C., Wakelin D. (1995). High levels of protection induced by a 40-mer synthetic peptide vaccine against the intestinal nematode parasite *Trichinella spiralis*. *Immunology*.

[29] Mcguire C., Chan W. C., Wakelin D. (2002). Nasal immunization with homogenate and peptide antigens induces protective immunity against *Trichinella spiralis*. *Infection and Immunity*.

[30] Pompa-Mera E. N., Yepez-Mulia L., Ocana-Mondragon A., García-Zepeda E. A., Ortega-Pierres G., González-Bonilla C. R. (2011). *Trichinella spiralis*: Intranasal immunization with attenuated *Salmonella enterica* Carrying a gp43 antigen-derived 30mer epitope elicits protection in BALB/c mice. *Experimental Parasitology*.

[31] Sette A., Fikes J. (2003). Epitope-based vaccines: an update on epitope identification, vaccine design and delivery. *Current Opinion in Immunology*.

[32] Skwarczynski M., Toth I. (2016). Peptide-based synthetic vaccines. *Chemical Science*.

[33] Bekaii-Saab T., Wesolowski R., Ahn D. H. (2019). Phase I immunotherapy trial with two chimeric HER-2 B-cell peptide vaccines emulsified in Montanide ISA 720VG and nor-MDP adjuvant in Patients with advanced solid tumors. *Clinical Cancer Research*.

[34] Tam J. P. (1988). Synthetic peptide vaccine design: synthesis and properties of a high-density multiple antigenic peptide system. *Proceedings of the National Academy of Sciences of the United States of America*.

[35] Moreno C. A., Rodriguez R., Oliveira G. A. (1999). Preclinical evaluation of a synthetic *Plasmodium falciparum* MAP malaria vaccine in Aotus monkeys and mice. *Vaccine*.

[36] Tallima H., Montash M., Veprek P., Velek J., Jezek J., el Ridi R. (2003). Differences in immunogenicity and vaccine potential of peptides from *Schistosoma mansoni* glyceraldehyde 3-phosphate dehydrogenase. *Vaccine*.

[37] Immanuel C., Ramanathan A., Balasubramaniyan M. (2017). Immunoprophylaxis of multi-antigen peptide (MAP) vaccine for human lymphatic filariasis. *Immunologic Research*.

[38] Mahajan B., Berzofsky J. A., Boykins R. A. (2010). Multiple antigen peptide vaccines against *Plasmodium falciparum* malaria. *Infection and Immunity*.

[39] Shreewastav R. K., Ali R., Uppada J. B., Rao D. N. (2012). Cell-mediated immune response to epitopic MAP (multiple antigen peptide) construct of LcrV antigen of *Yersinia pestis* in murine model. *Cellular Immunology*.

[40] Sahay B., Aranyos A. M., Mishra M. (2019). Immunogenicity and efficacy of a novel multi-antigenic peptide vaccine based on cross-reactivity between feline and human immunodeficiency viruses. *Viruses*.

[41] Edelman R., Wasserman S. S., Kublin J. G. (2002). Immediate-type hypersensitivity and other clinical reactions in volunteers immunized with a synthetic multi-antigen peptide vaccine (PfCS-MAP1NYU) against *Plasmodium falciparum* sporozoites. *Vaccine*.

[42] Scholzen A., Richard G., Moise L. (2019). Promiscuous *Coxiella burnetii* CD4 epitope clusters associated with human recall responses are candidates for a novel T-cell targeted multi-epitope Q fever vaccine. *Frontiers in Immunology*.

[43] Ma J., Wei Y., Zhang L. (2018). Identification of a novel linear B-cell epitope as a vaccine candidate in the N2N3 subdomain of *Staphylococcus aureus* fibronectin-binding protein A. *Journal of Medical Microbiology*.

[44] Mcmanus D. P., Loukas A. (2008). Current status of vaccines for schistosomiasis. *Clinical Microbiology Reviews*.

[45] Stephenson R., You H., Mcmanus D. P., Toth I. (2014). Schistosome vaccine adjuvants in preclinical and clinical research. *Vaccines*.

[46] Bethony J., Loukas A., Smout M. (2005). Antibodies against a secreted protein from hookworm larvae reduce the intensity of hookworm infection in humans and vaccinated laboratory animals. *The FASEB Journal*.

[47] Bethony J. M., Simon G., Diemert D. J. (2008). Randomized, placebo-controlled, double-blind trial of the Na-ASP-2 hookworm vaccine in unexposed adults. *Vaccine*.

[48] Ortega-Pierres G., Vaquero-Vera A., Fonseca-Linan R., Bermúdez-Cruz R. M., Argüello-García R. (2015). Induction of protection in murine experimental models against *Trichinella spiralis*: an up-to-date review. *Journal of Helminthology*.

[49] Loukas A., Bethony J., Brooker S., Hotez P. (2006). Hookworm vaccines: past, present, and future. *The Lancet Infectious Diseases*.

[50] Shulman Z., Gitlin A. D., Targ S. (2013). T follicular helper cell dynamics in germinal centers. *Science*.

[51] Fazilleau N., Mark L., Mcheyzer-Williams L. J., McHeyzer-Williams M. G. (2009). Follicular helper T cells: lineage and location. *Immunity*.

[52] Aljurayyan A., Puksuriwong S., Ahmed M. (2018). Activation and induction of antigen-specific T follicular helper cells play a critical role in live-attenuated influenza vaccine-induced human mucosal anti-influenza antibody response. *Journal of Virology*.

[53] Harker J. A., Lewis G. M., Mack L., Zuniga E. I. (2011). Late interleukin-6 escalates T follicular helper cell responses and controls a chronic viral infection. *Science*.

[54] Narazaki M., Kishimoto T. (2018). The two-faced cytokine IL-6 in host defense and diseases. *International Journal of Molecular Sciences*.

[55] Kishimoto T. (2010). IL-6: from its discovery to clinical applications. *International Immunology*.

[56] Mastelic G. B., Eberhardt C. S., Auderset F. (2015). MF59 mediates its B cell adjuvanticity by promoting T follicular helper cells and thus germinal center responses in adult and early life. *The Journal of Immunology*.

[57] Hollister K., Chen Y., Wang S. (2014). The role of follicular helper T cells and the germinal center in HIV-1 gp120 DNA prime and gp120 protein boost vaccination. *Human Vaccines & Immunotherapeutics*.

[58] Moon J. J., Suh H., Li A. V., Ockenhouse C. F., Yadava A., Irvine D. J. (2012). Enhancing humoral responses to a malaria antigen with nanoparticle vaccines that expand Tfh cells and promote germinal center induction. *Proceedings of the National Academy of Sciences of the United States of America*.

[59] Pilkinton M. A., Nicholas K. J., Warren C. M. (2017). Greater activation of peripheral T follicular helper cells following high dose influenza vaccine in older adults forecasts seroconversion. *Vaccine*.

[60] Iwasaki A., Medzhitov R. (2015). Control of adaptive immunity by the innate immune system. *Nature Immunology*.

[61] Ugolini M., Gerhard J., Burkert S. (2018). Recognition of microbial viability via TLR8 drives T_FH_ cell differentiation and vaccine responses. *Nature Immunology*.

[62] Linterman M. A., Pierson W., Lee S. K. (2011). Foxp3^+^ follicular regulatory T cells control the germinal center response. *Nature Medicine*.

[63] Sage P. T., Tan C. L., Freeman G. J., Haigis M., Sharpe A. H. (2015). Defective TFH cell function and increased TFR cells contribute to defective antibody production in aging. *Cell Reports*.

[64] Linterman M. A., Hill D. L. (2016). Can follicular helper T cells be targeted to improve vaccine efficacy?. *F1000Research*.

[65] Amodio D., Cotugno N., Macchiarulo G. (2018). Quantitative multiplexed imaging analysis reveals a strong association between immunogen-specific B cell responses and tonsillar germinal center immune dynamics in children after influenza vaccination. *Journal of Immunology*.

[66] Sakaguchi S., Yamaguchi T., Nomura T., Ono M. (2008). Regulatory T cells and immune tolerance. *Cell*.

[67] Ilic N., Gruden-Movsesijan A., Sofronic-Milosavljevic L. (2012). *Trichinella spiralis*: shaping the immune response. *Immunologic Research*.

[68] Finney C. A., Taylor M. D., Wilson M. S., Maizels R. M. (2007). Expansion and activation of CD4^+^CD25^+^ regulatory T cells in *Heligmosomoides poly*gyrus infection. *European Journal of Immunology*.

[69] Wen X., He L., Chi Y. (2011). Dynamics of Th17 cells and their role in *Schistosoma japoni*cum infection in C57BL/6 mice. *PLoS Neglected Tropical Diseases*.

[70] Baumgart M., Tompkins F., Leng J., Hesse M. (2006). Naturally occurring CD4^+^Foxp3^+^ regulatory T cells are an essential, IL-10-independent part of the immunoregulatory network in *Schistosoma mansoni* egg-induced inflammation. *Journal of Immunology*.

[71] Mcsorley H. J., Harcus Y. M., Murray J., Taylor M. D., Maizels R. M. (2008). Expansion of Foxp3^+^ regulatory T cells in mice infected with the filarial parasite *Brugia malayi*. *The Journal of Immunology*.

[72] Sun X.-M., Guo K., Hao C.-Y., Zhan B., Huang J.-J., Zhu X. (2019). *Trichinella spiralis* excretory–secretory products stimulate host regulatory T cell differentiation through activating dendritic cells. *Cells*.

[73] Holanda R. A., Munoz J. E., Dias L. S. (2017). Recombinant vaccines of a CD4^+^ T-cell epitope promote efficient control of *Paracoccidioides brasiliens*is burden by restraining primary organ infection. *PLOS Neglected Tropical Diseases*.

[74] Hassert M., Wolf K. J., Schwetye K. E., DiPaolo R., Brien J. D., Pinto A. K. (2018). CD4^+^T cells mediate protection against Zika associated severe disease in a mouse model of infection. *PLoS Pathogens*.

